# The 5000 Babies Project: Early detection of cerebral palsy movement patterns in infants

**DOI:** 10.21203/rs.3.rs-9856805/v1

**Published:** 2026-07-27

**Authors:** Lindsay Alfano, Patrick Tinsley, Marissa Koscielski, Megan Iammarino, Madalynn Wendland, Adrian Rodriguez, Maggie Dugan, Kathleen Adderley, Leah Lumbaca, Lindsay Pietruszewski, Kayla Nguyen, Garey Noritz, Linda P Lowes

**Affiliations:** The Abigail Wexner Research Institute at Nationwide Children’s Hospital; The Ohio State University College of Medicine; AngelEye Health; University of Cincinnati College of Medicine; University of Texas Health Science Center San Antonio; Cleveland State University; AngelEye Health; The Abigail Wexner Research Institute at Nationwide Children’s Hospital; The Ohio State University College of Medicine; Nationwide Children’s Hospital; The Abigail Wexner Research Institute at Nationwide Children’s Hospital; The Ohio State University College of Medicine; University of California, Los Angeles; AngelEye Health; Nationwide Children’s Hospital; The Abigail Wexner Research Institute at Nationwide Children’s Hospital; The Ohio State University College of Medicine

**Keywords:** early detection, cerebral palsy, infant pose estimation, data-centric AI

## Abstract

**Background:**

The prevalence of neuromotor conditions, such as cerebral palsy (CP), highlights a critical need for early and scalable detection tools. While expert-based assessments, like the General Movement Assessment (GMA), are effective in the high-risk neonatal population, the screening window and specialized training required of assessors limits widespread use for infants with or without newborn detectable risks. In an effort to address these limitations, this feasibility study explores the efficacy of using computer vision techniques to detect CP-related movements in infants between zero and six months corrected age.

**Purpose:**

The purpose of this study is to evaluate the feasibility of using computer vision and time series analysis of infant video data for the task of CP prediction.

**Methods:**

In this IRB-approved study, we prospectively collected video data on infants (0–6 months corrected age) and completed chart review at two years of age follow-up to identify children diagnosed with CP. Using an off-the-shelf pose estimation model, we calculated kinematic features such as torso rotation, distances between joints, and angular displacement. Deep learning models were trained on the data to classify infants based on a CP diagnosis at a two-year old follow-up. Model performance was evaluated in a cross-validation experimental set-up with an emphasis on ROC-AUC, sensitivity, and specificity metrics.

**Results:**

Out of the 930 infants with complete data, 42 were diagnosed with cerebral palsy at 2 years old. Models that were trained on the most informative kinematic features achieved median ROC-AUC of 0.82, median sensitivity of 0.81, and median specificity of 0.71. The most informative kinematic features were found to be those tracking the distances between two joints. Models trained on manually annotated, salient video segments consistently outperformed those trained on unannotated data.

**Conclusion:**

This feasibility study demonstrates the feasibility of applying pose-derived kinematic features and temporal deep learning models for early detection of cerebral palsy in infants. With further research, this approach may have potential to support clinicians with information for earlier screening and detection of aberrant movement patterns, ultimately improving long-term outcomes.

## Introduction

The prevalence of neuromotor conditions in newborns continues to increase, affecting approximately 16% of all children (1). Cerebral palsy (CP) is one of the more common causes of childhood disability and has a prevalence of 1.6/1000 live births (0.16%) (2). Although characterized by persistent movement and posture disturbances secondary to neurologic injury or malformation (3), the diagnosis of CP is often delayed to 18–24 months of age due to variations in “typical movement” and rates of development (3, 4). Evidence suggests that early detection and early intervention are associated with improved functional outcomes, language development, and quality of life for the patient and caregiver (5–10). With an infant’s greatest neuroplasticity window being within the first two years of life, facilitating early intervention maximizes the likelihood of an optimal outcome for the infant (6, 7, 10–13). Conversely, delayed detection reduces the opportunity to make a meaningful impact. Despite the evidence in the literature emphasizing the impact of early detection on clinical outcomes, universal early detection screening for cerebral palsy remains limited. Prechtl’s General Movement Assessment (GMA) is a non-invasive, observational examination that can help identify neonates at high risk for cerebral palsy. However, widespread implementation is often constrained due to specialized training requirements, recalibration, and resource allocation (14). To address these limitations, numerous research groups have attempted to develop algorithms to automate the GMA or predict CP using technology, such as wearable and noncontact sensor systems, available chart data, or computer vision.

The advent of complex sensor technology and artificial intelligence (AI) methodologies offers the potential for accessible and non-invasive screening tools in pediatric neurodevelopment. The capacity to quantitatively analyze infant movement patterns from standard video recordings presents a unique opportunity to overcome current barriers to trained human assessors for neuromotor assessments, especially when a later diagnosis is also known and supplied. Prior investigations have demonstrated the feasibility of employing computer vision to provide insights into neurologic health. However, further research and development are needed to distinguish typical versus atypical movement patterns both in infants at a higher risk for developing CP such as prematurity and for the general population through well infant check ups. The objective of this study is to report preliminary findings from experiments involving prospectively collected infant videos towards the prediction of a cerebral palsy diagnosis using time series analysis and computer vision models.

## Data Collection:

### Study Design

As part of the *5000 Babies Project* launched at the Abigail Wexner Research Institute at Nationwide Children’s Hospital, infants between 0 and 6 months corrected age were enrolled in an IRB-approved, prospective video collection study at academic pediatric hospitals and affiliate locations between October 2020 and September 2023. This study received ethical approval from Nationwide Children’s Hospital Institutional Review Board (approval ID “STUDY00000913”) on 09/09/2020. The approved data collection protocol and methods were carried out in accordance with relevant guidelines and regulations; written informed consent was obtained from a parent or legal guardian prior to enrollment in this study.

Also at the time of enrollment, age, prenatal/birth history, height/length, weight, and a list of any known diagnoses were collected. During the recording session, the infant was positioned supine on a mat under the camera-based study system. Using a computer interface, a video recording was captured for between four and six minutes at a frame rate of up to 30 frames per second (FPS). If the infant became fussy or could not tolerate the video, the recording was stopped; if the infant could tolerate the video after resting, feeding, or caregiver soothing, the recording was reattempted. Any enrolled infant who was unable to tolerate a video recording for the minimum of four minutes was excluded from this feasibility analysis.

At 2- and 3-years old, a retrospective chart review was completed for each infant. Infants without available medical records at these follow-up points (post video recording) were also excluded from this feasibility analysis.

All diagnoses or genetic markers that were associated with potential neuromotor features were labeled by condition name and listed as “Atypical.” Infants without a known diagnosis or displaying no developmental delay were categorized as “Typical.”

### Video Saliency Annotation

An internal team of human clinical annotators were tasked with marking the timepoints of the most informative 90 seconds in each video that supported the associated class label. Time periods were selected to optimize the amount of movement and behavioral state of the infant. We first eliminated any period where the view of the infant was obstructed by another person reaching into the frame, typically to calm the infant. We then selected the 90 seconds of the video in which the child was actively moving and preferably in a calm alert state; although sections in which the child was crying could be included if necessary. The corresponding pose estimates from these selected 2700 frames were the primary source of data in this work. 90% of videos listed the 2700 frames as one contiguous segment, while the remainder of videos listed two separate segments of frames as salient in the decision-making process.

### Patient Demographics

Following informed consent, 940 infants, between 0 and 6 months corrected age, were enrolled in our prospective video collection study across 38 clinics within the Nationwide Children’s Hospital ecosystem of hospital and affiliate sites. Of the 940 infants, 930 infants had salient video recordings and follow-up data, and were thus included in this study. Ten infants were excluded due to the infant being too irritated or too distracted by external stimuli (objects or humans) to complete the attempted recording.

657 infants did not have a neuromotor diagnosis at the 2 or 3 year old timepoint and were categorized as “Typical,” or NKD (“No Known Diagnosis”). The remainder of enrolled infants (273) were categorized as “Atypical,” with 42 such patients having a positive cerebral palsy (CP) diagnosis. In the case where a sample had multiple diagnosis labels, if CP was listed at all, the sample was labeled as CP. The sample size of 42 was treated as the positive class for this feasibility analysis. Demographic attributes for the infants included in this case study can be seen in **Table 1**. Infants born before 37 weeks qualified as preterm.

The datasets used and/or analysed during the current study available from the corresponding author on reasonable request.

## Methodology

### Infant Pose Estimation

In accordance with the guidelines and regulations stipulated in the work’s IRB approval, top-down videos of supine infants were collected via an Azure Kinect camera for between four and six minutes. Capture angle for the camera sensor remained consistent across all collection sites, and lighting conditions were also held as consistent as possible. Pose estimates were extracted for all videos included in the study. Infant pose estimation was accomplished with the OpenMMLab Pose Estimation Toolkit (15), specifically the RTMPose-m model (16). In addition to visual inspection of the pose estimates overlaid on the original videos, the findings from Jahn et al. (17) and Gama et al. (18) supported our decision to not fine-tune the model and use the pre-trained pose estimate model for our collection of videos. The pose estimate model tracked 17 keypoints over the course of each video. In this study, only 12 were used for training our Typical-versus-CP classification model. The 5 keypoints on the face (nose, eyes, ears) were excluded, although future research may re-examine their relevance in the task of CP prediction. The 12 keypoints used were the left and right wrists, elbows, shoulders, hips, knees, and ankles.

### Data Preprocessing

In order to allow proper comparison of infants of varying size and positioning within the frame, the extracted pose estimate data was centered around the infant’s pelvis and scaled to have unit torso length of 1.0. The pose estimates were also rotated such that the torso was aligned exactly vertically along the y-axis i.e., with the pelvis center at (x = 0, y = 0), and the shoulder center at (x = 0, y = 1). This torso rotation angle was recorded as a feature (“rotation_angle”) and used in downstream experiments. In order to reduce noise from pose estimate data, the time series were also smoothed with the Savitzky-Golay filtering with window size 11 and degree 2.

### Feature Engineering

After smoothing, centering, and rotating the original pose estimate data for all videos, a comprehensive collection of kinematic features, each represented by time series, was constructed with the goal of identifying informative features when predicting CP. Time series capturing asymmetry were calculated by subtracting right-associated values from left. Consequently, in these asymmetry-related time series, values closer to zero suggest more symmetric, concurrent motion across the body, while non-zero values indicate asymmetric motion; positive values favor the left side of the body, while negative values favor the right. 12 positional and computational feature sets were extracted, and are defined in Table 2. Additionally, 7 more feature sets that encompassed permutations of the previous 12 feature sets were tested; their labels and constituents are defined in Table 3.

### Model Training

Features were engineered using preprocessed pose estimates for all 930 infants included in this feasibility analysis. Samples (consisting of features and corresponding class label) were then served as input to a deep learning model. Samples were 2700 frames long and were labeled either as salient or not, as judged by our clinical team’s annotations. The saliency of input samples was used primarily to determine whether models trained on video data that humans deemed relevant outperform models trained on non-salient video snippets.

Due to its ability to capture long-range temporal dependencies, the chosen model backbone was the InceptionTime model (19), as implemented in the tsai Python package (20).

For both the salient case and non-salient case, 20 models were trained for each of the 19 possible input feature sets described above (for a total of 380 trained models). For each input feature set, the 20 models were randomly initialized and trained on a different subset of Typical samples. This was done in order to ensure robust results when dealing with our severe class imbalance (657 Typical samples versus 42 CP samples). Each of the 20 cross-validation trials included an initial random resampling of 42 samples from the negative (Typical) class. For each trial, a TimeInception model was trained on 75% of the class-balanced set of samples, and validated on the remaining 25% of samples.

### Model Evaluation

Models were evaluated in the 20-fold cross-validation setup previously described in order to properly assess model generalizability. The chosen evaluation criterion was the ROC-AUC score, which quantifies how well the model can separate two classes: in our case, Typical and CP. Additionally, given the clinical nature of the task of CP prediction, sensitivity and specificity are also reported in the next section.

## Results

**Figure 1:**
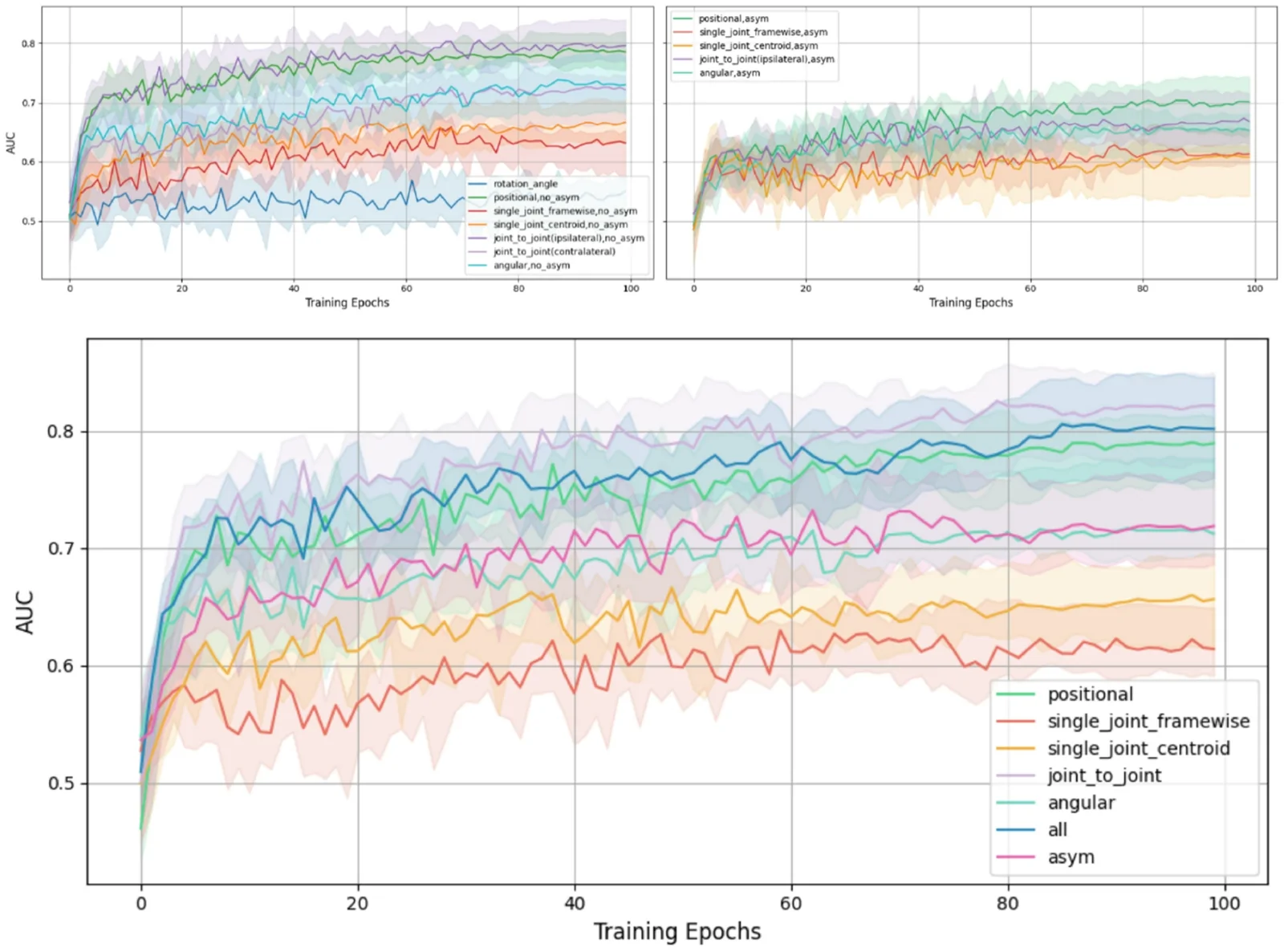
(top row) Model performance (AUC) during the training process for feature sets that do not include asymmetry (left) and do include asymmetry (right); (bottom row) model performance during training for the feature sets that combine previously defined feature sets. Training features were generated from video snippets marked as salient by our clinical team.

**Figure 2:**
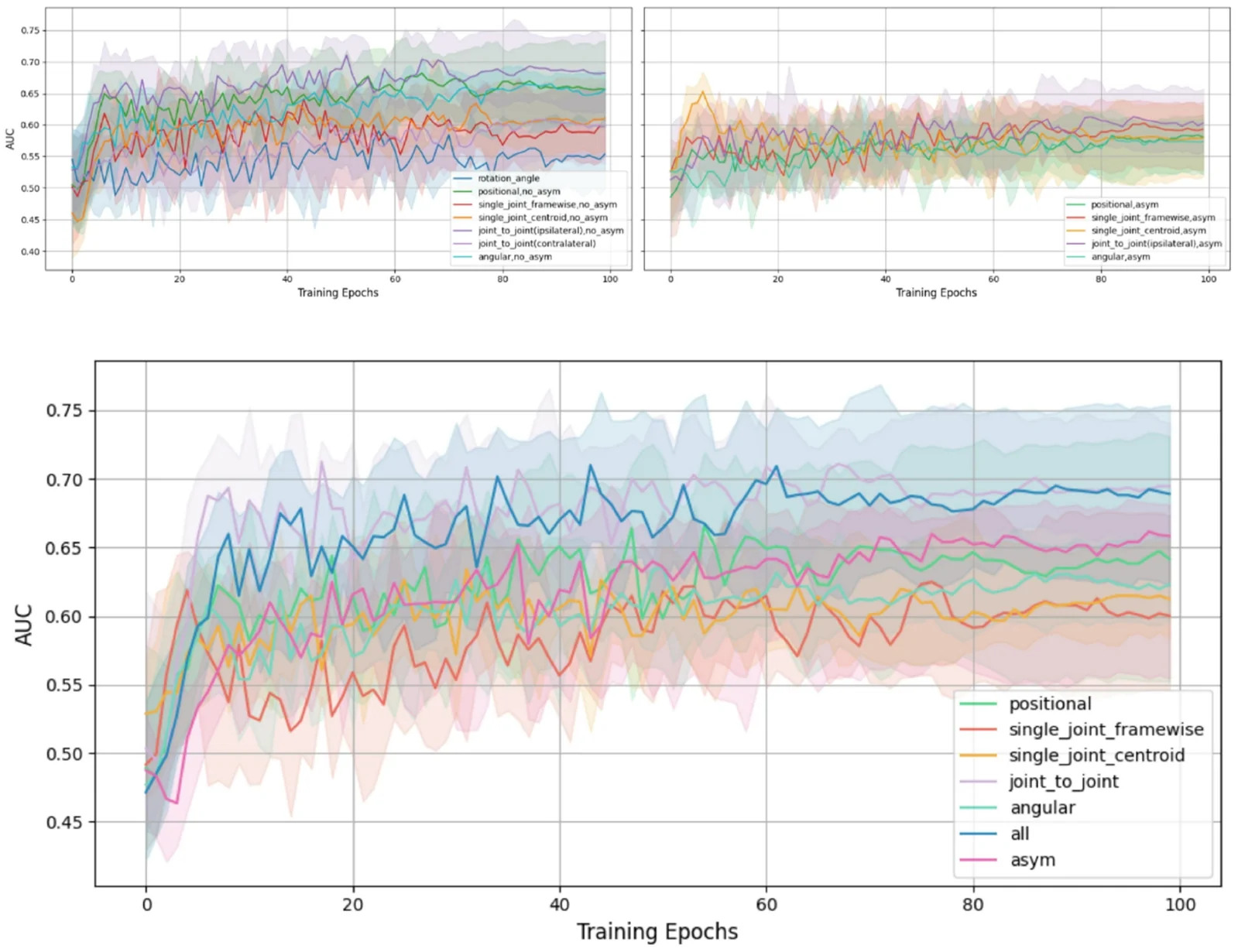
(top row) Model performance (AUC) during the training process for feature sets that do not include asymmetry (left) and do include asymmetry (right); (bottom row) model performance during training for the feature sets that combine previously defined feature sets. Training features were generated from video snippets **not** marked as salient by our clinical team.

Figures 1 and 2 compile the model performance for all models during their respective training process for salient samples and non-salient samples, respectively. Each banded line tracks the median and interquartile range (IQR) for the AUC metric for the 20 models trained on a given feature set.

In the salient sample case, the highest performing median ROC (0.82) was achieved when training on the “joint_to_joint” features, suggesting that distances between multiple keypoints and their evolution over time is most informative when predicting CP, especially ipsilateral pairs of keypoints on the same side of the body, namely: wrist to ankle, elbow to knee, wrist to shoulder, and ankle to hip.

**Figure 3:**
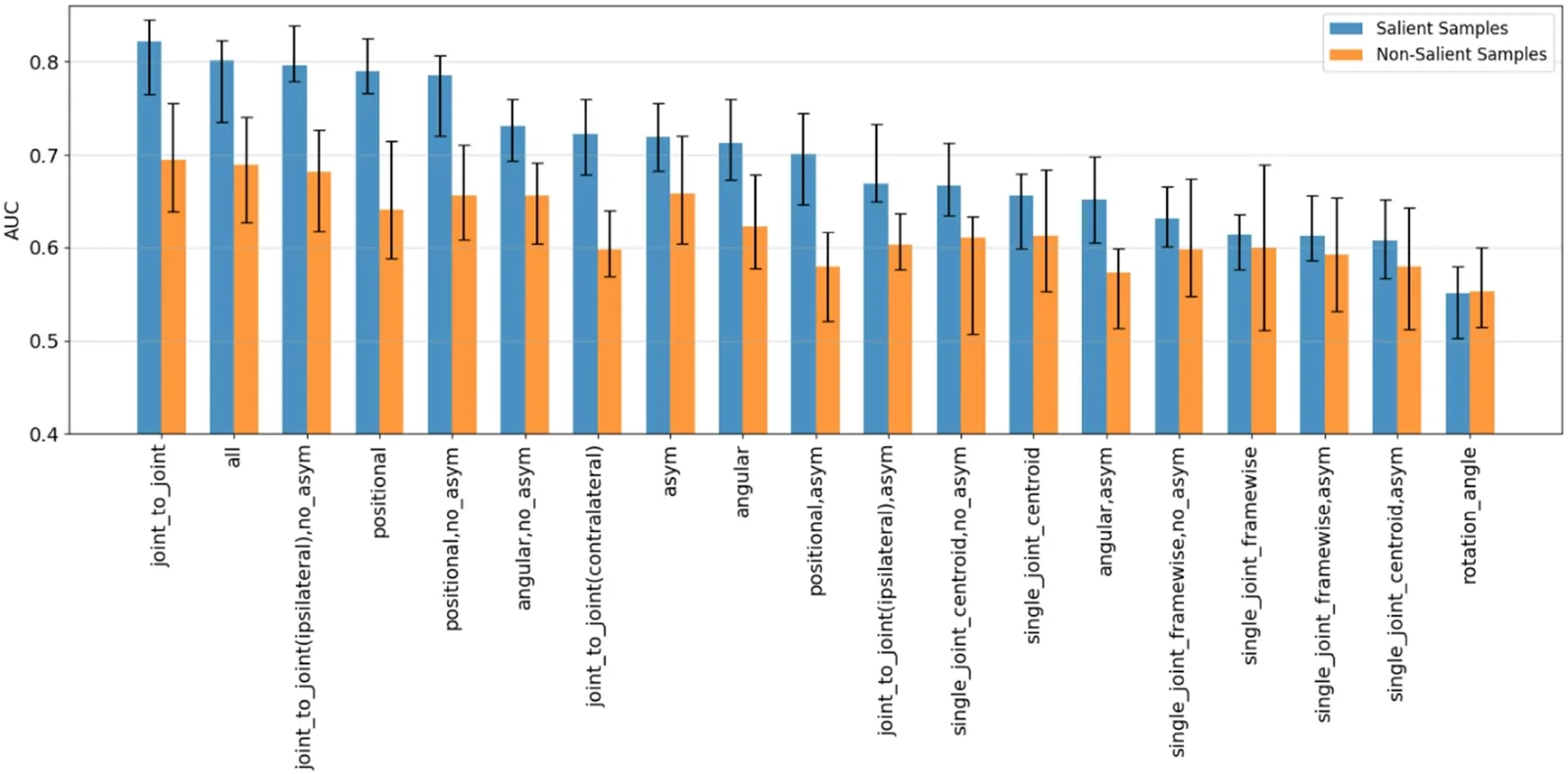
Model performance (AUC) during cross-fold validation for features generated from salient video snippets (blue/left) and non-salient video snippets (orange/right).

In the non-salient sample case, the models trained on “joint_to_joint” features again yielded the highest median ROC (0.69). However, model performance with non-salient samples was significantly worse than the salient case across the board, as reflected in Fig. 3.

Models trained on single keypoint-related features did not match the performance of models trained on pairs of keypoints; this is apparent in the lower performance of “single_joint_framewise” and “single_joint_centroid” in the bottom rows of Figs. 1 and 2. The top rows of Figs. 1 and 2 also show that (generally) asymmetry-related features do not provide additional predictive power compared to asymmetry-unrelated time series. This was the case for both salient and non-salient testing scenarios.

**Figure 4:**
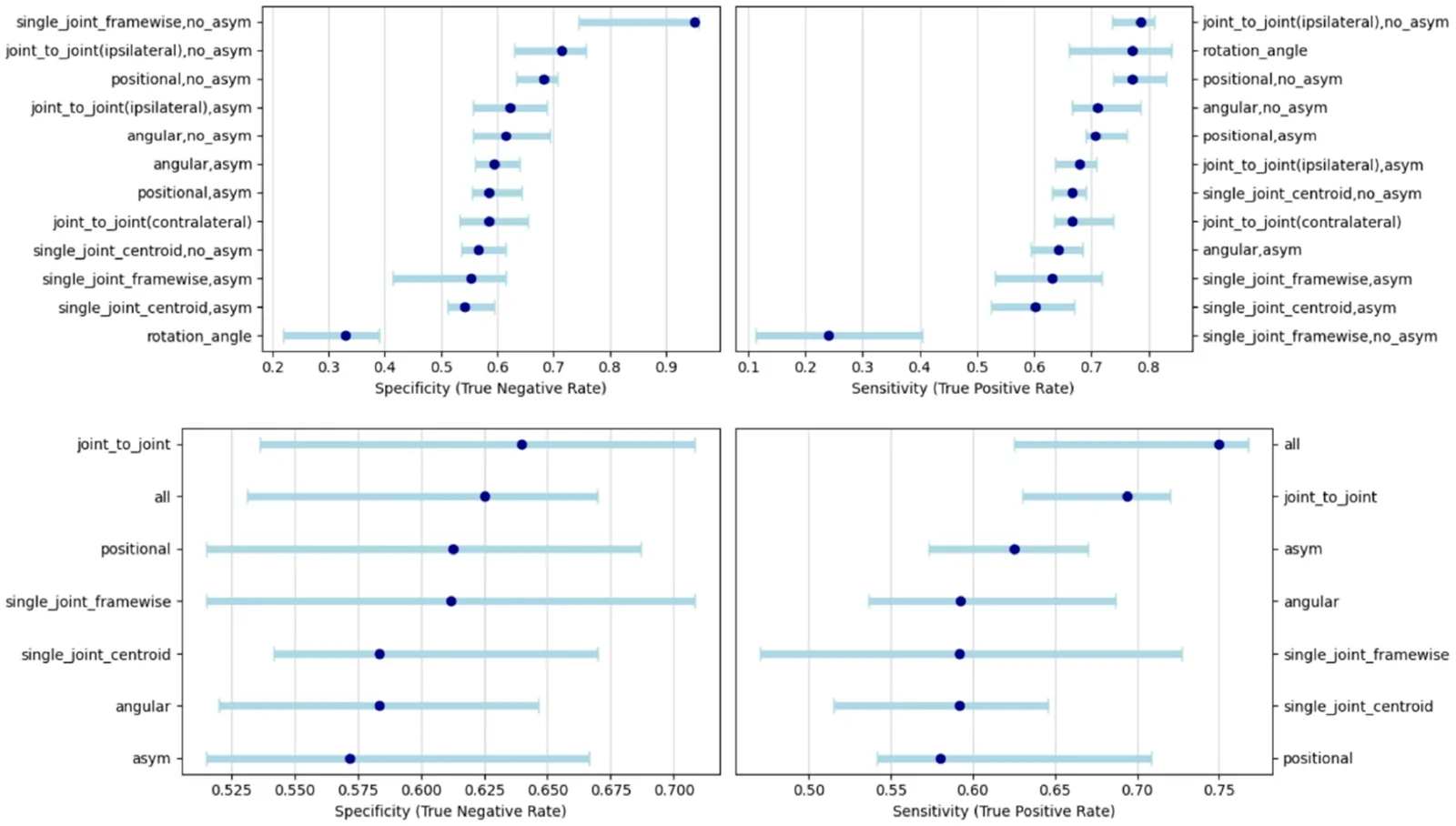
Forest plots of model specificity (left) and model sensitivity (right) for models trained on video snippets marked as salient, broken down by pre-defined features (top row), and combining features (bottom row).

Figure 4 shows the sensitivity and specificity achieved by these same models, sorted by performance. An interesting observation is the performance of models trained only on a single feature: “rotation_angle.” As seen in the forest plots, these models showed high sensitivity and low specificity, meaning the models consistently predicted CP, producing a high number of false positives. On the other end, the “single_joint_framewise, no_asym” models showed high specificity and low sensitivity. These models consistently predicted Typical, leading to an abundance of false negatives. Results in ROC (Figs. 1,2,3) show that neither of these models achieved proper class separation, leading us to believe that these features are not informative in the task of CP prediction.

The top row of Fig. 4 does however support the case for models trained “joint_to_joint (ipsilateral), no_asym” features, with the highest-achieving median specificity and sensitivity: 0.71 and 0.78, respectively. The bottom row of Fig. 4 (relative to the top row) discourages the combination of features, even joint-to-joint features, and highlights the strength of models trained on ipsilateral keypoints or positional data alone.

## Discussion

This feasibility analysis used modern computer vision techniques to assess the feasibility of using video data in the task of predicting cerebral palsy. With an off-the-shelf pose estimation pipeline, the construction of handcrafted features, and an off-the-shelf deep learning model, a median ROC of 0.82 was achieved with high median specificity (0.71) and median sensitivity (0.78).

The findings from our experiments illustrate that time series AI-based detection of cerebral palsy from pose-derived features is feasible, with joint-to-joint features and raw positional data providing the strongest predictive value. In contrast, some feature sets (including torso rotation and single joint-related time series) were less effective, suggesting that tracking individual keypoints over time is not sufficient for reliable CP prediction and may be excluded in subsequent studies. Asymmetry- and angular-related features offered mixed benefits. While not universally advantageous, both features could be paired with multi-keypoint or positional features for improved class separation and predictive power. We also demonstrated that the identification of salient motion intervals is valuable to the model development pipeline, as models trained on salient video snippets consistently outperformed those trained on non-salient sequences. This finding, though not entirely unexpected, reinforced our hypothesis that annotated samples would be more valuable throughout the model training process. Given this result, future work will address the task of automatically identifying salient video snippets in longer video streams.

The gold standard for the early detection and intervention of high risk infants for conditions such as cerebral palsy relies on expert human-based assessors (21–23). Financial, staffing, and training hurdles subsequently limit the number of available assessors for visual assessments, such as the General Movements Assessment. However, despite its proven efficacy, the traditional GMA has significant limitations. Although cramped synchronized movement patterns (often categorized as rigid, repetitive, and lacking fluidity across limbs and trunk) are considered strong early predictors of CP, the clinical implications of poor repertoire movements remain unclear. Additionally, the GMA requires extensive training and recalibration to achieve reliable accuracy. This reliance on “gestalt perception” and a high degree of training makes it difficult to scale the GMA internationally. Despite its strong clinical performance, studies have found the GMA accuracy drops to nearly 50% without rigorous assessor recalibration. Furthermore, the GMA is not a definitive biomarker. Cerebral palsy continues to be a clinical diagnosis rather than based on a definitive test. This suggests that an earlier accurate diagnosis could require a qualified clinician that may not exist in all communities. There is a need for both a broader application of the GMA for early screening of infants as well as research and development of neuromotor screening tools for infants that do not fall into the GMA window, term infants, and infants with no known risk factors.

A growing body of research suggests that artificial intelligence and machine learning can overcome these limitations by providing a more objective, quantitative, and scalable approach to neuromotor assessment. By using computer vision to track anatomical landmarks from video data, these methods can quantify movement features such as velocity, frequency, and amplitude, which are then used by ML algorithms to predict clinical outcomes. A recent study by Feng et al., reported a pose model that was correlated with the level of consciousness within a NICU setting (24). Using a pressure sensor mat, a study by Maitre et al., reported high sensitivity and specificity to identify Cramped Synchronized Movement Patterns within the writhing window. Another study by McCay et al., reported a GMA-feature classification system in neonates with strong sensitivity and specificity (25). While these studies report encouraging results to support early detection and intervention, to our knowledge our feasibility analysis is the first that has demonstrated feasibility in a sample of both low and high risk infants who develop cerebral palsy by 2 years of age.

Our study is not without limitations. Despite cross-validation mitigating the small sample size, data scarcity remains an obstacle, underscoring the need for larger and more diverse patient populations. Future work with a larger sample size should assess model generalizability, explore multimodal data integration, and extend to multiple diagnoses in broader clinical settings.

## Conclusion

This feasibility study addresses the feasibility and success of applying pose-derived kinematic features and deep learning for early detection of cerebral palsy in infants. Our results indicate that joint-to-joint features are particularly valuable in discriminative power, while individual joint-based tracking contributes minimally. Additionally, we see marked performance gains with clinician-annotated video snippets, highlighting the need for thoughtful data selection in model development. The limited number of positive samples in this study will be expanded in future work as we develop machine learning models to detect and describe a more encompassing set of aberrant movement patterns.

## Supplementary Material

Tables are available in the Supplementary Files section.

Supplementary Files

This is a list of supplementary files associated with this preprint. Click to download.

- Tables.docx

## Figures and Tables

**Figure 5:**
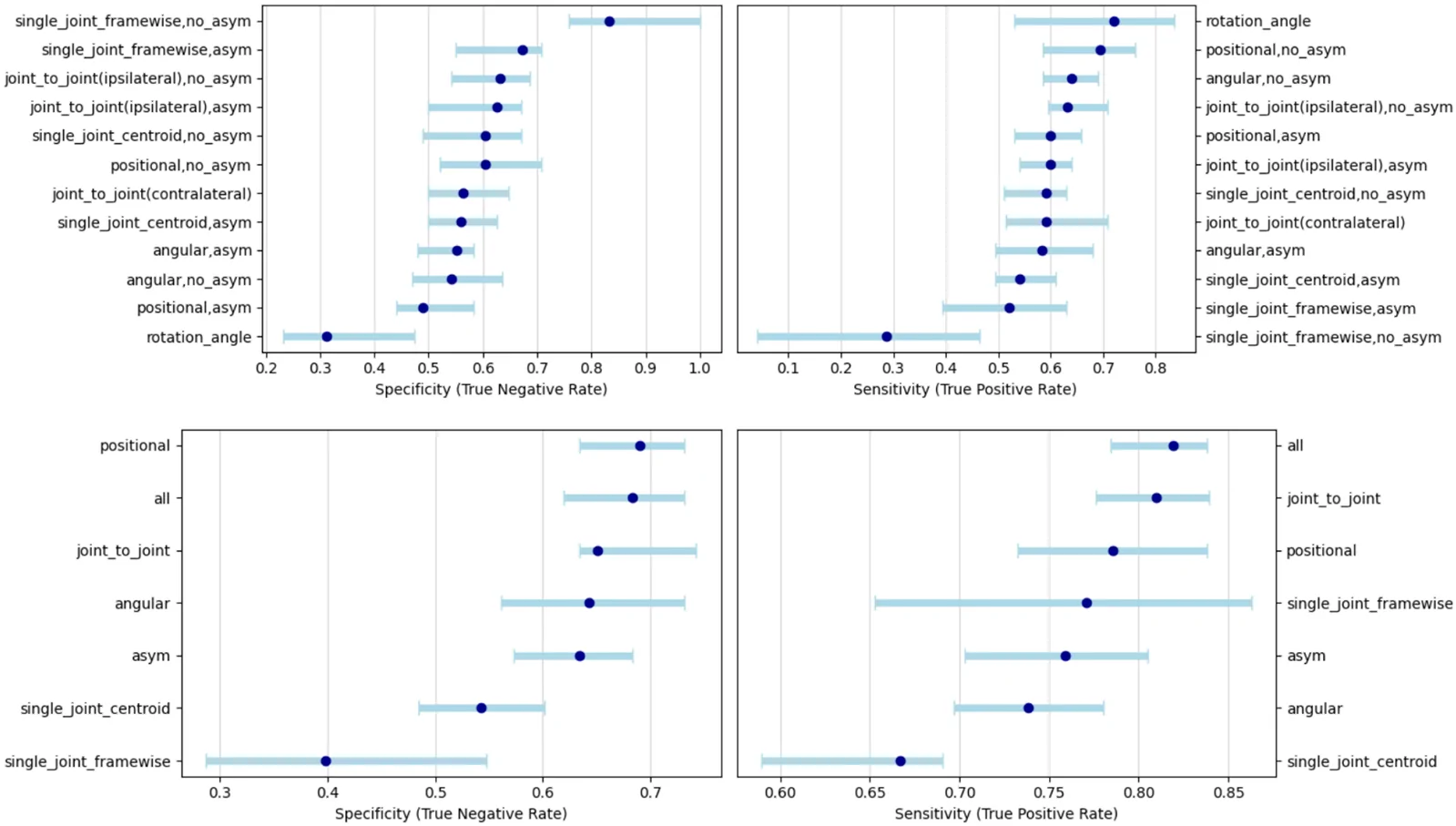
Forest plots of specificity (left) and sensitivity (right) for models trained on video snippets marked as non-salient, broken down by feature set.
